# Friendship and Consumption Networks in Adolescents and Their Relationship to Stress and Cannabis Use Intention

**DOI:** 10.3390/ijerph18073335

**Published:** 2021-03-24

**Authors:** María Cristina Martínez-Fernández, Cristina Liébana-Presa, Elena Fernández-Martínez, Lisa Gomes, Isaías García-Rodríguez

**Affiliations:** 1 SALBIS Research Group, Faculty of Health Sciences, Campus de Ponferrada, Universidad de León, 24401 Ponferrada, Spain; mmartf@unileon.es; 2 SALBIS Research Group, Faculty of Health Sciences, Universidad de León, 24071 León, Spain; elena.fernandez@unileon.es; 3 Nursing School, Minho University, 4704-553 Braga, Portugal; lgomes@ese.uminho.pt; 4 SECOMUCI Research Groups, Department of Electric, Systems and Automatics Engineering, Universidad de León, 24071 León, Spain; isaias.garcia@unileon.es

**Keywords:** cannabis, adolescents, stress, social network analysis, network, friendship

## Abstract

Background: Cannabis is an illegal psychoactive substance that’s use is widespread among adolescents. During adolescence, many changes can cause stress. In this phase, the group of friends becomes increasingly important, being a situation of vulnerability for the beginning of cannabis use, either as an escape mechanism or due to peer’s influence. Therefore, the purpose of this study is to describe and analyze the structure of the consumption and friendship network, the intention to use cannabis, and the stress in a secondary school class. Methods: An online platform with validated self-reported questionnaires were used for data collection. Results: The sample consisted of adolescents (*n* = 20) aged 14–16 from a third-year class of compulsory secondary education in Ponferrada (León, Spain). Significant differences were obtained concerning consumption intention and the different network metrics in both the friendship and consumption networks. Subsequently, the representation of these networks was carried out. Conclusions: Social Network Analysis is a very useful tool that provides a picture of the context in which adolescents are located. In the consumption network, there are central actors who have not yet consumed cannabis; this is a crucial moment to implement prevention strategies.

## 1. Introduction

According to the World Health Organization (WHO), cannabis is the most widely consumed drug among young people in 2018; approximately 4.7% of young people aged 15 to 16 years had consumed it at least once [1]. In Spain, the most recent data indicate that cannabis is the illegal psychoactive substance with the highest prevalence of use, with an average age of onset of 14.8 years; in fact, 398,600 students aged 14 to 18 years had consumed cannabis during 2016 [2]. Substance use is related to multiple risk factors, including school failure and problematic behaviors [3]. Additionally, substance use can lead to physical, psychological and social disorders that demand the design of effective prevention policies [4]. Substance use and abuse is, therefore, one of the main risk factors for health, where adolescent users are more likely to manifest social and personal issues, lower psychological adjustment and emotional competence [5].

Adolescence is a period of change. A key characteristic of youth is that it is a relevant phase in the consolidation, gain or loss of previously acquired habits and lifestyles, which has an impact on the future health status of individuals [6]. Emotional and behavioral adjustment problems have been seen as mediators between cannabis use and psychosis risk [7]. Early initiated cannabis use results as a significant marker of mental health and behavioral risk [8]. Therefore, well-being in adolescents plays a key role in preventing substance use [7]. Moreover, burnout experienced by adolescents in high school can have an influence on cannabis use, being relevant to the consideration of academic stress in the prevention of cannabis use, since its use causes a loss of academic expectations and motivation [9].

As previously mentioned, cannabis use may appear as an escape mechanism from stress [10]; in fact, those adolescents who do not use cannabis cope with stress in a more flexible way [11]. There are cognitive schemes based on beliefs of grandiosity and insufficient self-control that are significantly associated with drug use [12]. The theory of planned behavior provides an explanation for cannabis use and describes the relationship between cognitive characteristics of individuals and the development and maintenance of behavioral patterns [13]. Developmental theories indicate that the transition to adolescence is decided by an increase in the frequency of peer interactions, the adoption of more sophisticated interpersonal behaviors, new social roles and experiences, adolescents’ motivation to develop a stable sense of identity, and young people’s reliance on peer feedback (and their perceived status by peers) [14].

In this context, the concept of Social Network Analysis (SNA) emerged, is proving an impact on the health. Peers are fundamental in the social organization of adolescents, as they are present in the academic environment and activities. Likewise, during adolescents’ development, they increasingly acquire more autonomy and independence from their family environment [15,16]. SNA has been widely applied in public health, social support, social capital, influences on health behaviors, and the social structure of information dissemination [17]. Many studies have been carried out using the SNA in adolescents, indicating how the position in the peer group and popularity have an influence on relevant aspects, such as leisure activities and eating behaviors that spread in the friendship network, influencing overweight [18] and even sleeping habits, where the most popular adolescents sleep less than the rest of the individuals in the network [19].

SNA is a theoretical and methodological paradigm that makes possible to evaluate the relational context empirically and to capture contexts of social interaction, which determine the behavior of the actors that are part of that context [17]. Thus, one of the key components in adolescent well-being are social networks. The literature has found that having friends or being connected to friendship networks that exhibit risky behaviors (smoking or drinking alcohol) implies an increased risk of engaging in these behaviors, both initially and over time [20]. SNA can be helpful in better understanding the mechanisms underlying the connection between friendships and risk behaviors [20]. Adolescent contacts are important for the establishment of adolescent health, as well as for the acquisition and maintenance of risk behaviors [21,22].

Peer influence is defined as a phenomenon characterized by the presence of both selection and socialization. By understanding why adolescents fit in with their peers, it is possible to develop preventive measures that alternatively address the psychological motivations that lead to conformity at present [14]. However, peer influence from adolescence to adulthood has been found to be later much less clear [23].

Following the exposed problem, this study has the following question: what is the relationship between the structural characteristics of friendship and cannabis use networks, an individual’s intention to use cannabis and young adolescents’ stress? The SNA can become a useful tool that will allow us to know the behavioral pattern of the network in order to plan concrete and effective interventions on the identified risk behaviors (cannabis use intention and stress), in short, to promote healthy and sustainable networks. Therefore, the aim of this study is to describe the structure of the consumption and friendship network, the intention to use cannabis and the stress of young adolescents in a secondary school class, and, furthermore, to analyze the relationships between network structural variables, consumption intention and stress and to represent these relationships in order to identify peer leaders and plan interventions that promote health (less stress and less consumption).

## 2. Materials and Methods

A cross-sectional, descriptive and correlational study was conducted. A nonprobabilistic convenience sample was selected. The sample is composed of all students enrolled in a 3rd-year class in a Compulsory Secondary Education (E.S.O.) center in the city of Ponferrada (León, Spain) during the 2019/2020 academic year. The criteria for selecting this class are twofold: (i) the age of the students is at the age of initiation of cannabis use and (ii) previous academic courses that ensure the coexistence of students. The selected class has 20 students.

### 2.1. Variables and Measuring Instruments

Stress: Stress was measured using the Student Stress Inventory–Stress Manifestations (SSI–SM) [24] validated in Spanish for adolescents [25]. This questionnaire consists of 22 items, with a five-point Likert-type scale (not at all, rarely, sometimes, often and totally). These items are distributed in three factors: emotional (α = 0.79), physiological (α = 0.62) and behavioral (α = 0.66).

Cannabis use: To measure cannabis use, the Spanish Survey on Drug Use in Secondary Education (ESTUDES) [2] is used. This questionnaire is composed of different items, according to the substances, from which we selected those referring to the block of cannabis use. The section is made up of 10 items related to the time that cannabis has been used, the first time it was used and how it is used.

Cannabis Use Intention: The validated Cannabis Use: Intention Questionnaire (CUIQ) was used for the youth population [26]. This questionnaire comprises 12 items. Each one of the items is evaluated by means of a Likert-type scale from 1 to 5 points.

Networks: To determine the classroom consumption and friendship network, a limited census of actors is used that is matched to the list of classroom peers. Students are asked to nominate only those actors/peers in their class with whom they would go out to consume. With the data collected, a sociocentric matrix is obtained. In the same way, to obtain the classroom friendship network, students were asked to nominate from the census of actors those classmates with whom they share their free time. A 4-point Likert-type scale was used, where 0 means “I never share my free time,” and 4 means “We are always together.”

### 2.2. Procedure

Data were collected by online questionnaire. Authorization was obtained by the corresponding Compulsory Secondary Education center, and the teacher of the class involved was contacted to make them aware of the procedure. Data collection took place on different days in February 2020. The online questionnaire was carried out in different web programming languages, PHP (Zend, Minneapolis, MN, USA) and MySQL (Oracle, Santa Clara, CA, USA) for its dynamization, together with a front-end based on HTML5 (World Wide Web Consortium (W3C), Cambridge, MA, USA), CSS (World Wide Web Consortium (W3C), Cambridge, MA, USA), JavaScript (Oracle, Santa Clara, CA, USA) (and jQuery (The OpenJS Foundation, San Francisco, CA, USA)), complying with different standards and measures that facilitate its visualization on different devices (responsive design).

### 2.3. Data Analysis

Qualitative variables are shown as frequencies and percentages. Quantitative variables were expressed as mean and standard deviation. After verifying that the quantitative variables did not follow a normal distribution, using the Kolmogorov–Smirnov test with Lilliefors correction, nonparametric correlations were performed, and Spearman’s rho coefficients were obtained. Statistical analyses were carried out using Statistical Package for the Social Sciences software (SPSS v. 26.0) (IBM, Armonk, NY, USA).

For network analysis, the data obtained were transferred to Excel and processed using the UCINET V 6.0 program and NetDraw [27]. Centrality measures (see Table 1) were calculated for the participants.

### 2.4. Ethical Considerations

The anonymity and confidentiality of the study subjects were considered at all times. Being underage minors, prior authorization was received from their parents or legal guardians for participation in the study, as well as the informed consent of the participants. The data obtained from the research will be treated in accordance with both the Constitutional Law 3/2018, of December 5, on the Protection of Personal Data and Guarantee of Digital Rights and the General Data Protection Regulation of the European Union EU 2016/679 (GDPR). In addition, permission was requested from the educational center and the competent body for education in the region (Consejería de Educación de la Junta de Castilla y León). The study was approved by the ethics committee (ETICA-ULE-035-2019) of the University of León (Spain), which ensures compliance with ethical and legal aspects.

## 3. Results

The sample consisted of a total of 20 adolescents, of which 10% were female (*n* = 2), and 90% male (*n* = 18), with an age measurement of 14.45 ± 0.61 (min = 14; max = 16).

First, regarding the results of cannabis use, the data obtained place the prevalence of cannabis use at 10% (*n* = 2). Of these, one individual’s last cannabis use was 40 days ago, while the other shows a more habitual use, being the last use 3 days ago. The age of onset of use was 13 years old. One of the two consumers used cannabis mixed with tobacco. When asked that, if cannabis consumption were legal would they consume it, 20% of the students said that they would consume it, while 15% of the sample had already tried it (including consumers). Finally, it is worth noting that one student consumed cannabis alone quite often, while the other indicated that he had never consumed cannabis alone. Regarding the cannabis use intention, the total values are 1.35 ± 1.13; however, we find a high maximum, that result in the equivalence table indicates that it is above the 90th percentile. This means that 90% of young people of the same age have a lower intention to consume than this one.

Secondly, the descriptive results obtained for the variable stress and are set out in Table 2. Stress obtained total values of 37.65 ± 18.45.

Thirdly, Table 3 shows the values of the structure of the consumption and friendship networks, the actors who may be in more central positions in the networks and with greater degree of influence. The following parameters are described: indegree, outdegree, degree of proximity (out/in closeness), betweenness, and influence through the eigenvector. It is found that networks are similar in terms of values and density, which may suggest that adolescents select their friends as the peers with whom they would go out to consume.

**Figure 1:**
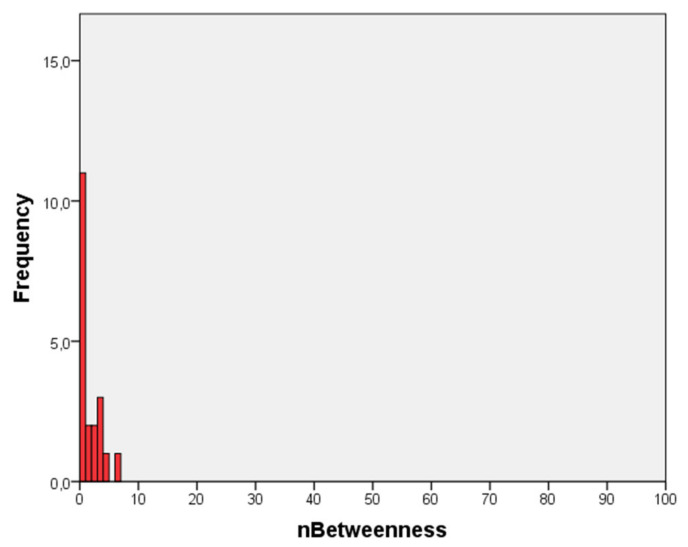
Normalized Betweenness (nBetweenness) centrality distribution.

Figure 1 illustrates the distribution of normalized betweenness in the friendship network, showing that a large number of individuals have a very low intermediation capacity. In fact, 30% of the individuals have no intermediation capacities at all.

A correlational analysis between the different variables, stress, cannabis use intention and friendship and consumption networks is shown in Table 4.

No significant correlations were found for cannabis use with these data, given the small sample size of the consumers. Behavioral manifestations of stress involve behaviors, such as acting defensively, neglecting friendships or showing negative attitudes in different interpersonal relationships. In this regard, it is noteworthy that a statistically significant correlation was found between the behavioral manifestations of stress dimension and the metrics of out closeness (r = 0.541), outdegree (r = 0.530) and the degree of betweenness (r = 0.496). In addition, statistically significant correlations were found in this network for in closeness (r = −0.511), emotional manifestations and physical manifestations and outdegree (r = 0.446). The cannabis use intention appears statistically significantly correlated with different network metrics: for the friendship network, outdegree (r = 0.596), out closeness (r = 0.531) and betweenness (r = 0.598), while, in the consumption network, outdegree (r = 0.252), out closeness (r = 0.500) and betweenness (r = 0.549). On the other hand, it shows a statistically significant correlation with total stress (r = 0.622) and its different manifestations: emotional (r = 0.510), physiological (r = 0.687) and behavioral (r = 0. 0479).

**Figure 2:**
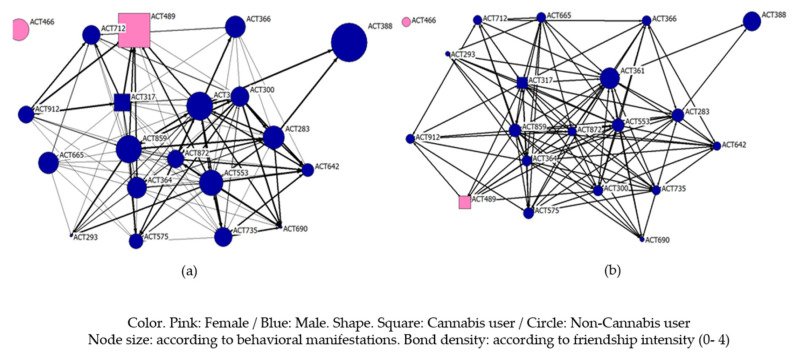
Friendship network structure and the study variables; (**a**) total of Student Stress Inventory–Stress Manifestations, (**b**) bbehavioral of Student Stress Inventory–Stress Manifestations.

Figure 2 represents the classroom friendship network, where males are represented with a blue color and females with a pink color. Cannabis users are identified as square-shaped nodes and nonusers as circles. The size of the nodes varies according to their total stress. The intensity of the relationships is measured through the strength of the ties (0–4), where a greater strength indicates that these students are united by a bond of friendship by spending a large part of their free time together. The size of the nodes varies as a function of the total stress scores. Thus, we find how individuals who are consumers are close and could be considered friends. These nodes, although they have relationships with those actors who are more central, establish ties of greater intensity with actors who are on the periphery. However, it stands out how the girl who uses cannabis establishes ties of friendship with the central actors, being, in addition, one of the individuals with higher levels of stress. In general, we found a dense network, where relationships of varying intensity were high. We found an isolated node and a node on the periphery that only maintains a relationship with two individuals from the others in the class, being also one of the individuals with the highest level of general stress.

**Figure 3:**
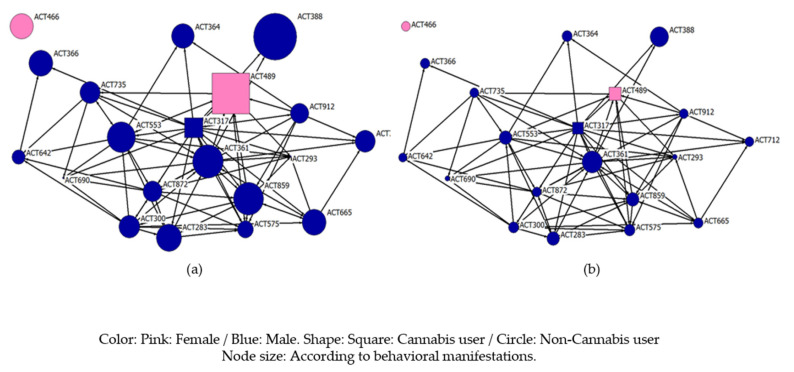
Consumption network structure and the study variables; (**a**) total of Student Stress Inventory–Stress Manifestations, (**b**) behavioral of Student Stress Inventory–Stress Manifestations.

Figure 3 shows the classroom consumption network from which students were asked to select, out of all their classmates, those individuals with whom they would consume. As in the previous case, the size of the nodes varies as a function of the total stress scores or the behavioral manifestations of stress. The structure is similar to the friendship network, since the central actors are maintained, in this case, the central actor being a cannabis user. Consequently, it may be an indication that the friendship network influences cannabis consumption, being that this consumption is accepted by those nodes with whom they share more time.

**Figure 4:**
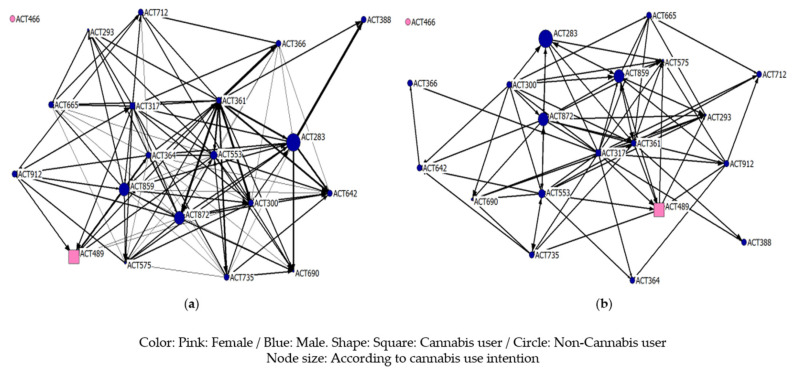
Cannabis use intention (**a**) consumption network; (**b**) friendship network.

Figure 4 shows the two networks, but, in this case, the size of the node varies depending on the cannabis use intention. Although most of the subjects obtained scores according to the mean, we found nodes of greater size, whose scores were indicative of an intention higher than normal. These actors are found both in the periphery and in the center of the network and are individuals in whom action must be taken through different intervention strategies when they are in a situation of risk at the beginning of cannabis use.

## 4. Discussion

The aim of this article is to describe the consumption and friendship network of adolescents and to relate it to stress in its different manifestations and the cannabis use intention. Subsequently, the representation of the friendship and consumption network is carried out for those variables in which significant results have been obtained: the intention to use cannabis, total stress and behavioral manifestations of stress, which are related to acting defensively, neglecting friendships, talking more about peers and teachers, picking on others, etc.

SNA allows us to locate those individuals with positions of influence within the network. Although multiple factors influence cannabis use, the literature indicates that family stressors have a direct impact on the progression to problematic cannabis use, as well as their consequent indirect effects through the school experience of young people [30]. Furthermore, cannabis use causes difficulties in school performance, creating a lack of motivation and interest that feeds back by increasing cannabis use [9]. In adolescence, there is a change in the relationship system of young people, from focusing on the family to associating with peers; in this sense, the SNA helps to explain personal attachment to the community from a local level in the structure of the friendship network and supports the peer influence theory [31]. Previous experiences have employed the SNA in school settings with adolescents to identify those who are considered leaders among their peers and train them to deliver e-cigarette prevention programs to the rest of the class [32]. In this case, through SNA, we have identified the most central actors, two individuals who are in central positions in the consumption network, who may be in positions of risk of initiation, problematic consumption or ability to transmit these behaviors to the rest of their peers, and through whom to carry out prevention strategies and interventions customized for each setting.

In addition, it is necessary to consider the emotional and behavioral adjustment problems that mediate the relationship between cannabis use and risk of psychosis [7], as this study points out, highlighting the importance of prevention by focusing on the mediating role of emotional and behavioral problems to train young people in socioemotional competencies in school contexts [7]. The literature highlights the importance of prevention and addressing adolescents before actual cannabis use takes place; as [33] says, adolescents who think more positively about being under the influence of marijuana, those with greater approval from their social environment, and those with less confidence in their ability to abstain from using have a greater intention to initiate marijuana use [33]. In addition, adolescents will have a more favorable attitude toward drug use if their contacts with environments and inciting companies and the contacts with drugs maintained by friends are greater [34]. Thus, SNA can be used as an intervention strategy to promote behavioral changes, since networks influence the health of their members by generating a context with its own behavior and norms where members can influence each other through persuasion, information exchange or support [35]. In this sense, the correlation found in this study between the betweenness centrality metric and the intention to use cannabis could be a potential danger, regarding the spreading of this unhealthy habit, as the individuals with higher betweenness values play an important role in the dissemination of behavior through the networks. This is especially relevant if one takes into account that 30% of the individuals have no intermediation capacities in the network.

This study presents advances in the area of cannabis use, since it is the first study to analyze the friendship and consumption network, stress and cannabis use intention. However, it has some limitations that should be considered: This is a small sample, which is not representative of the population, and the results should be interpreted with caution. In addition, there is a possibility that some participants did not declare their consumption, as can be seen in the classroom consumption network with very central actors not consuming, where it can be seen that those who have consumed are very central, but not all of them. In addition, in future studies, it would be useful to carry out longitudinal designs that can indicate how these variables behave over time and be able to carry out causal explanations, as well as designs where health education interventions are proposed to these population groups.

## 5. Conclusions

SNA is a useful tool that provides a picture of the context where adolescents are located. It allows identifying those that are more isolated, which is considered a disadvantage, and the most popular, who can be chosen as role models by their peers. After knowing the consumption network of the class, we find central actors who that have not initiated cannabis consumption. This indicates a crucial moment to carry out prevention strategies adapted to each school context. These strategies should be implemented at earlier ages, since we found several cannabis-consuming students who may have been influenced by their peers.

## Figures and Tables

**Table 1:** Definitions of the Social Network Analysis (SNA) centrality metrics.

Concept	Definition
InDegree Centrality	Number of people who ask the actor for advice [28].
Betweenness centrality	Extent to which an actor serves as a potential “go-between” for other pairs of actors in the network by occupying an intermediary position on the shortest paths connecting other actors [28].
Closeness centrality	Measures how close an actor is to the other actors in the network [29].
Eigenvector Centrality	Measure of actor centrality that takes into account the centrality of the actors to whom the focal actor is connected. Thus, an actor whose three friends have many connections will have higher Eigenvector centrality than an actor whose three friends have few connections [28].

**Table 2:** Descriptive statistics of stress.

Questionnaire		*n*	Min–Max	M ± SD	Me
SSI–SM	Emotional	20	0–40	18.85 ± 9.99	18
	Physiological	20	0–17	9.15 ± 4.46	9
	Behavioral	20	0–25	9.65 ± 5.93	8.5
	Total	20	0–77	37.65 ± 18.45	37.5

Note: SSI–SM; Student Stress Inventory–Stress Manifestations. Min–Max; Minimum–Maximum. M; Mean. SD; Standard Deviation. Me; Median.

**Table 3:** Descriptive indicators of centrality in consumption and friendship networks.

SNA Centrality Metrics		Min–Max.	M ± SD
Consumption Network	Indegree	0–0.42	0.22 ± 0.18
	Outdegree	0–0.89	0.22 ± 0.25
	Out closeness	0.25–0.83	0.43 ± 0.18
	In closeness	0.25–0.41	0.37 ± 0.03
	Betweenness	0–11.41	1.84 ± 3.20
	Eigenvector	0–54.26	28.38 ± 14.30
Friendship Network	Indegree	0–0.30	0.20 ± 0.20
	Outdegree	0–0.63	0.20 ± 0.10
	Out closeness	0.25–0.83	0.54 ± 0.21
	In closeness	0.25–0.51	0.45 ± 0.06
	Betweenness	0–6.39	1.46 ± 1.83
	Eigenvector	0–53.87	28.43 ± 14.21

Note: Min–Max; Minimum–Maximum. M; Mean. SD; Standard Deviation.

**Table 4:** Correlations between centrality metrics of friendship and consumption networks, stress and cannabis use intention.

			SSI–SM				
			Emotional	Physiological	Behavioral	Total	CUIQ
Consumption Network	Indegree	Rho	−0.354	−0.150	0.191	−0.088	−0.034
	Outdegree	Rho	−0.024	0.191	0.376	0.192	0.525 *
	Out closeness	Rho	−0.015	0.174	0.346	0.182	0.500 *
	In closeness	Rho	−0.272	−0.181	0.084	−0.121	−0.249
	Betweenness	Rho	0.048	0.265	0.421	0.278	0.549 *
	Eigenvector	Rho	−0.302	0.014	0.394	0.007	0.285
Friendship Network	Indegree	Rho	−0.328	0.021	0.125	-0.50	0.265
	Outdegree	Rho	0.138	0.446 *	0.530 *	0.401	0.596 **
	Out closeness	Rho	0.181	0.414	0.541 *	0.399	0.531 *
	In closeness	Rho	−0.511 **	-0.188	0.074	−0.270	0.016
	Betweenness	Rho	0.153	0.417	0.496 *	0.375	0.598 **
	Eigenvector	Rho	−0.295	0.194	0.297	0.063	0.337
SSI–SM. Total		Rho	0.825 **	0.925 **	0.799 **		
CUIQ		Rho	0.510 *	0.687 **	0.479 *	0.622 **	

Note. CUIQ; Cannabis Use: Intention Questionnaire. SSI–SM; Student Stress Inventory–Stress Manifestations. Rho: Spearman’s correlation. * Correlation is significant at the 0.05 level. ** Correlation is significant at the 0.01 level.

## Data Availability

Not Applicable.

## References

[1] World Health Organization Adolescent Mental Health. https://www.who.int/es/news-room/fact-sheets/detail/adolescent-mental-health.

[2] Observatorio Español de las Drogas y las Adicciones ESTUDES 2018/19. Encuesta sobre el Uso de Drogas en Enseñanzas Secundarias en España (1994–2018). http://www.pnsd.mscbs.gob.es/profesionales/sistemasInformacion/sistemaInformacion/pdf/ESTUDES_2018-19_Informe.pdf..

[3] Espada J.P., Griffin K.W., Botvin G.J., Méndez X. (2003). Adolescencia: Consumo de Alcohol y Otras Drogas. Papeles Del Psicólogo.

[4] Palacio A.B., Santana J.D.M., Monroy M.F., Sánchez I.G., Meneses G.D. (2012). Modelo Explicativo Del Comportamiento De Los Jóvenes Ante El Botellón Y El Cannabis Desde La Perspectiva Del Marketing Social. Rev. Española Investig. Mark ESIC.

[5] Extremera N., Fernández-Berrocal P. (2014). The Subjective Happiness Scale: Translation and Preliminary Psychometric Evaluation of a Spanish Version. Soc. Indic. Res..

[6] Fernández Villa T., Alguacil Ojeda J., Ayán Pérez C., Bueno Cavanillas A., Cancela Carral J.M., Capelo Álvarez R., Delgado Rodríguez M., Jiménez Mejías E., Jiménez Moleón J.J., Llorca Díaz J. (2013). UNIHCOS Project: Dynamic cohort of Spanish college students to the study of drug and other addictions. Rev. Esp. Salud. Publica..

[7] Fonseca-Pedrero E., Lucas-Molina B., Pérez-Albéniz A., Inchausti F., Ortuño-Sierra J. (2019). Experiencias Psicóticas Atenuadas y Consumo de Cannabis En Adolescentes de La Población General. Adicciones.

[8] Hawke L.D., Wilkins L., Henderson J. (2020). Early cannabis initiation: Substance use and mental health profiles of service-seeking youth. J. Adolesc..

[9] Walburg V., Dany M., Aurélie M. (2015). Burnout among High-School Students and Cannabis Use, Consumption Frequencies, Abuse and Dependence. Child Youth Care Forum.

[10] Low N.C.P., Dugas E., O’Loughlin E., Rodriguez D., Contreras G., Chaiton M., O’Loughlin J. (2012). Common stressful life events and difficulties are associated with mental health symptoms and substance use in young adolescents. BMC Psychiatry.

[11] Kruczek A. (2017). Mood and Coping Flexibility in a Group of Adolescents Using Marijuana. Alcohol. Drug Addict. Alkohol. Narkom..

[12] Calvete E., Estévez A. (2019). Consumo de Drogas En Adolescentes: El Papel Del Estrés, La Impulsividad y Los Esquemas Relacionados Con La Falta de Límites. Adicciones.

[13] Ajzen I. (1991). The Theory of Planned Behavior. Organ. Behav. Hum..

[14] Brechwald W.A., Prinstein M.J. (2011). Beyond Homophily: A Decade of Advances in Understanding Peer Influence Processes. J. Res. Adolesc..

[15] Moody J. (2001). Peer Influence Groups: Identifying Dense Clusters in Large Networks. Soc. Netw..

[16] Osgood D.W., Feinberg M.E., Wallace L.N., Moody J. (2014). Friendship Group Position and Substance Use. Addict. Behav..

[17] Luke D.A., Stamatakis K.A. (2012). Systems Science Methods in Public Health: Dynamics, Networks, and Agents. Annu. Rev. Public Health.

[18] De La Haye K., Garry P.M., Carlene W. (2010). Obesity-Related Behaviors in Adolescent Friendship Networks. Soc. Netw..

[19] Li X., Kawachi I., Buxton O.M., Haneuse S., Onnela J.P. (2019). Social Network Analysis of Group Position, Popularity, and Sleep Behaviors among U.S. Adolescents. Soc. Sci. Med..

[20] Jeon K.C., Goodson P. (2015). US Adolescents’ Friendship Networks and Health Risk Behaviors: A Systematic Review of Studies Using Social Network Analysis and Add Health Data. PeerJ.

[21] De la Haye K., Green H.D., Kennedy D.P., Pollard M.S., Tucker J.S. (2013). Selection and Influence Mechanisms Associated with Marijuana Initiation and Use in Adolescent Friendship Networks. J. Res. Adolesc..

[22] Tucker J.S., de la Haye K., Kennedy D.P., Green H.J., Pollard M.S. (2014). Peer Influence on Marijuana Use in Different Types of Friendships. J. Adolesc. Health.

[23] Pollard M.S., Tuckera J.S., Greena H.D., de la Hayeb K., Espelagec D.L. (2018). Adolescent Peer Networks and the Moderating Role of Depressive Symptoms on Developmental Trajectories of Cannabis Use. Addict. Behav..

[24] Fimian M.J., Philip A., Fastenau J.H., Tashner A., Cross H. (1989). The Measure of Classroom Stress and Burnout among Gifted and Talented Students. Psychol. Sch..

[25] Escobar M., Blanca M.J., Fernández-Baena F.J., Trianes M.V. (2011). Adaptación Española de La Escala de Manifestaciones de Estrés Del Student Stress Inventory (SSI-SM). Psicothema.

[26] Lloret D., Morell-Gomis R., Laguia A., Moriano J.A. (2018). Design and Validation of a Cannabis Use Intention Questionnaire (CUIQ) for Adolescents. Adicciones.

[27] Borgatti S.P., Everett M.G., Freeman L.C. (2002). Ucinet 6 for Windows: Software for Social Network Analysis.

[28] Kilduff M., Tsai W. (2003). Social Networks and Organizations.

[29] Borgatti S.P., Everett M.G. (1997). Network Analysis of 2-Mode Data. Soc. Netw..

[30] Butters J.E. (2002). Family stressors and adolescent cannabis use: A pathway to problem use. J. Adolesc..

[31] Chang C.Y., Wu C.I. (2020). The friend influence in network neighbourhood context on adolescents’ community attachment. Int. J. Adolesc. Youth.

[32] Chu K.H., Sidani J., Matheny S., Rothenberger S.D., Miller E., Valente T., Robertson L. (2021). Implementation of a cluster randomized controlled trial: Identifying student peer leaders to lead E-cigarette interventions. Addict. Behav..

[33] Malmberg M., Overbeek G., Vermulst A.A., Monshouwer K., Vollebergh W.A.M., Engels R.C.M.E. (2012). The theory of planned behavior: Precursors of marijuana use in early adolescence?. Drug Alcohol. Depend..

[34] Jiménez M.V.M., Díaz F.J.R., Ruiz C.S. (2006). Factores relacionados con las actitudes juveniles hacia el consumo de alcohol y otras sustancias psicoactivas. Psicothema.

[35] Knox J., Schneider J., Greene E., Nicholson J., Hasin D., Sandfort T. (2019). Using social network analysis to examine alcohol use among adults: A systematic review. PLoS ONE.

